# Incidence and risk factors of neonatal infections in a rural Bangladeshi population: a community-based prospective study

**DOI:** 10.1186/s41043-018-0136-2

**Published:** 2018-03-09

**Authors:** Dipak K. Mitra, Luke C. Mullany, Meagan Harrison, Ishtiaq Mannan, Rashed Shah, Nazma Begum, Mamun Ibne Moin, Shams El Arifeen, Abdullah H. Baqui, For the Projahnmo Study Group in Bangladesh

**Affiliations:** 1 School of Public Health, Independent University, Bangladesh (IUB), Dhaka, Bangladesh; 2 International Center for Maternal and Newborn Health, Department of International Health, Johns Hopkins Bloomberg School of Public Health, Johns Hopkins University, Baltimore, MD USA; 3 Save the Children, Bangladesh country office, Dhaka, Bangladesh; 4 Department of Global Health, Save the Children-USA, Washington, DC, USA; 5 International Centre for Diarrhoeal Disease Research, Dhaka, Bangladesh

**Keywords:** Neonatal infections, Risk factors, Bangladesh, Prospective study

## Abstract

**Background:**

Infections cause about one fifth of the estimated 2.7 million annual neonatal deaths worldwide. Population-based data on burden and risk factors of neonatal infections are lacking in developing countries, which are required for the appropriate design of effective preventive and therapeutic interventions in resource-poor settings.

**Methods:**

We used data from a community-based cluster-randomized trial conducted to evaluate the impact of two umbilical cord cleansing regimens with chlorhexidine solution on neonatal mortality and morbidity in a rural area of Sylhet District in Bangladesh. Newborns were assessed four times in the first 9 days of life by trained community health workers (CHWs) using a WHO IMCI-like clinical algorithm. Cumulative incidence of the first episode of infections in the first 9 days of life was estimated using survival analysis technique accounting for survival bias and competing risk of death before the occurrence of infection. A multivariable generalized estimating equation log-binomial regression model was used to identify factors independently associated with infections.

**Results:**

Between 2007 and 2009, 30,267 newborns who received at least one postnatal assessment visit by a CHW within the first 9 days of life were included in this study. Cumulative incidence of infections in the first 9 days of life was 14.5% (95% CI 14.1–14.9%). Significant risk factors included previous child death in the family [RR 1.10 (95% CI 1.02–1.19)]; overcrowding [RR 1.14 (95% CI 1.04–1.25)]; home delivery [RR 1.86 (95% CI 1.58–2.19)]; unclean cord care [RR 1.15 (95% CI 1.03–1.28)]; multiple births [RR 1.34 (95% CI 1.15–1.56)]; low birth weight [reference: ≥ 2500 g, RR (95% CI) for < 1500, 1500–1999, and 2000–2499 g were 4.69 (4.01–5.48), 2.15 (1.92–2.42), and 1.15 (1.07–1.25) respectively]; and birth asphyxia [RR 1.65 (1.51–1.81)]. Higher pregnancy order lowered the risk of infections in the study population [compared to first pregnancy, RR (95% CI) for second, third, and ≥ fourth pregnancy babies were 0.93 (0.85–1.02), 0.88 (0.79–0.97), and 0.79 (0.71–0.87), respectively].

**Conclusion:**

Neonatal infections and associated deaths can be reduced by identifying and following up high-risk mothers and newborns and promoting facility delivery and clean cord care in resource-poor countries like Bangladesh where the burden of clinically ascertained neonatal infections is high. Further research is needed to measure the burden of infections in the entire neonatal period, particularly in the second fortnight and its association with essential newborn care.

**Trial registration:**

NCT00434408. Registered February 9, 2007.

## Background

Globally, an estimated 2.7 million neonates (1–28 days) die every year, and approximately 98% of these deaths occur in developing countries [1, 2]. Although neonatal mortality is declining, the rate of such decline has been slower than that observed for postneonatal and 1 to 4-year-old child mortality [3, 4]. Thus, neonatal death is increasingly becoming more important as a proportion of under-five child deaths globally. In 2000, 38% of the under-five child deaths was due to neonatal deaths, and by 2013, this proportion became 44% [5, 6]. Infections including sepsis, meningitis, pneumonia, and tetanus are responsible for 22.2% of neonatal mortality globally and up to 50% of neonatal deaths in high mortality settings [7–9]. Neonatal deaths can be prevented with low-cost interventions at community and primary care facilities [10, 11]. It has been estimated that timely identification and management of serious infections can reduce 20 to 55% of neonatal deaths with 90% service coverage, and further reduction can be achieved in conjunction with additional antenatal and intrapartum care [10, 12]. Recent evidence demonstrating the effectiveness of simpler antibiotic regimens for management of newborn infections that can be delivered at the first-level health facilities has the potential to greatly expand access to care and reduce mortality [13, 14]. Data on burden and risk factors of community-acquired neonatal infections in developing countries are scant but are critically essential for designing and implementing targeted interventions in such settings [15]. Using data from a large community-based study that evaluated the impact of chlorhexidine cleansing of the umbilical cord on neonatal mortality and morbidity, this paper provides an estimate of incidence of clinically ascertained community-acquired neonatal infections in the first 9 days of life and identifies risk factors for infections in the first 9 days of life of neonates in a rural area of Bangladesh.

## Methods

### Study design and participants

This study uses observational data from a cohort of newborns and their mothers that participated in a community-based trial conducted in three rural sub-districts (Beanibazar, Zakigonj, and Kanaighat) of Sylhet District in Bangladesh. Detailed design, procedure, and major findings of the trial have been described elsewhere [16, 17]. Briefly, the study evaluated the impact of two regimens of umbilical cord cleansing (single application and 7-day application) with 4.0% chlorhexidine solution compared to dry cord care on overall neonatal mortality and incidence of cord infections (ClinicalTrials.gov identifier: NCT0043448). An estimated 546,000 population in 22 unions (the smallest administrative unit with ~ 25,000 populations with a first-level health facility) participated in the study. The area was divided into 133 clusters, each served by a female community health worker (CHW) and 4–5 village health workers (VHWs), who implemented the interventions and collected data.

### Study procedures

CHWs enumerated all households at the beginning of the study and made a list of married women of reproductive age (MWRA) including their pregnancy status in the study area. They continued two monthly home visits to update the list of MWRAs and to identify new pregnant women. All women identified as pregnant during the study period were invited to participate in the study. Those agreeing to participate provided data on age, parity, date of last menstrual period, occupation, literacy, complete birth history, and socio-economic information of the household.

CHWs delivered a package of maternal and neonatal health interventions to all enrolled women during the two antenatal home visits made at 12–16 weeks and at 32–34 weeks of pregnancy. The intervention package included a supply of iron and folic acid, a clean birthing kit, messages on birth and newborn care preparedness (BNCP), and advice on essential newborn care (clean cord care, breastfeeding, and thermal care), and postnatal danger signs [11, 17]. CHWs made six postnatal home visits scheduled on days 1, 3, 6, 9, 15, and 28–35 to deliver interventions and collect data. Physical assessment for signs of clinical infections was performed in the first four visits. All live births in the study areas that received at least one postnatal assessment visit by CHW in the first 9 days of life were included in this study.

### Training and quality assurance

All CHWs received in-house and competence-based training for 6 weeks under the direct supervision of trained physicians. The training sessions used a standard curriculum including skills development for behavior change communication, delivery of BNCP and essential newborn care, clinical assessment of neonates, and identification and management of sick newborns using the clinical algorithm. After the training, all CHWs were standardized for clinical assessment in Sylhet MAG Osmani Medical College Hospital, a tertiary care teaching hospital serving the study population. Field data quality was ensured through direct supervision by field supervisors. Periodic supervisory visits and standardization exercise sessions were organized to ensure data quality. Data forms were edited by supervisory staff for completeness, accuracy, and consistency. Data entry system was designed with built-in range and consistency checks. All identified incomplete or inconsistent data were verified in the field by senior project staff.

### Follow-up visits and data collection

#### Exposure variables

CHWs collected information on exposure variables during antenatal visits and the first postnatal visit using a set of questionnaires and assessment tools. Information on socio-demographic and economic variables (age at enrollment, educational status of women and their husbands, basic housing structure, sanitation and source of drinking water, household assets, religion, household size) and previous obstetric history were collected at enrollment. Data on antenatal care, consumption of iron tablets, TT immunization, and antenatal complications (history of fever, severe abdominal pain, swelling of hand, leg or face, vaginal bleeding, convulsion, severe headache, blurring of vision) were collected from all women during antenatal visits and the first postnatal visit. Information on delivery characteristics (date and time of birth, birth attendants, place of birth, prolonged labor, prolonged rupture of membrane, retained placenta, cord care), newborn characteristics (sex, birth weight, gestational age at delivery, conditions of the baby at birth), and essential newborn care (clean cord care, breastfeeding, thermal care) were collected on the first postnatal visit.

#### Outcome variable

The primary outcome of the study was neonatal infection during the first 9 days of life as clinically ascertained by the CHWs. During the postnatal visits scheduled on days 1, 3, 6, and 9, CHWs assessed all babies who were alive on the day of the visits and recorded signs of infections and other illnesses. While most visits happened as scheduled on days 1, 3, 6, and 9, in some cases, the visits were made in the intervals, so during the analysis stage, the actual age of newborns was calculated at the time of each visit for consistency. Assessment visits beyond day 9 are excluded from this analysis. Clinical infections were defined as the presence of any of the seven signs (panel 1) on the day of assessment; these signs included the seven signs of the WHO recommended Integrated Management of Childhood Illness (IMCI) algorithm [18].

Panel 1: Clinical signs used for CHW’s assessment in the study

- History of or observed convulsion
- Feeding difficulty confirmed by observation
- Respiratory rate 60 per minute or more
- Severe chest indrawing
- Temperature ≥ 37.5 °C
- Temperature ≤ 35.5 °C
- Does not move without stimulation

### Statistical analyses

We estimated the cumulative incidence of neonatal infections in the first 9 days of life and 95% confidence interval (CI) using a survival analysis technique. To minimize survival bias, we allowed late entry that babies entered the analysis at the age of first visit by CHWs and contributed person time until the occurrence of the first episode of infection, death, lost to follow-up, or reaching age day 9. Death before the occurrence of infection was considered a competing risk and was adjusted in the estimation procedure, as a conventional Kaplan-Meier estimator is likely to overestimate cumulative incidence in the presence of a competing risk such as death [19]. We used Stata command “stcompet” written by Enzo Coviello for this purpose [20].

Risk factor analysis was done using a log-binomial regression with a log link function and binomial family. A generalized estimating equation (GEE) approach with exchangeable correlation structure was used to adjust for the clustered nature of the data [21, 22]. The CHW working area was considered as the cluster in our analysis due to variation in case detection proportion by CHWs. In case of convergence failure with log-binomial model, a Poisson regression with a robust standard error was used [23]. Missing data of covariates were imputed using the “hotdeck” method by cluster [24].

The exposure variables were grouped into (1) socio-demographic and household factors (maternal age, birth order, parental educational status, household crowding, previous history of child deaths, household economic status), (2) maternal factors (obstetric history, antenatal care, delivery characteristics), and (3) newborn factors (sex, gestational age, birth weight, condition of the baby at birth, essential newborn care). For measuring socio-economic status, a categorical wealth index variable was created using information on ownership of durable household assets, source of drinking water, type of latrine, and characteristics of dwelling house using principal component analysis [25]. In our final model, variables related to the initiation of breastfeeding, oil massage, and delaying bathing the baby were excluded as early morbidities are likely to negatively influence these newborn care practices [26]. Data analyses were done in Stata (version 12) statistical software [27].

## Results

**Fig. 1:**
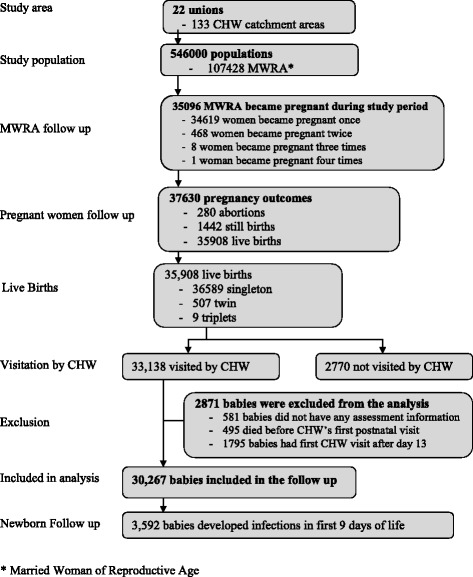
Study profile

Between June 2007 and September 2009, 35,908 live births were recorded in the study population. CHWs made at least one postnatal home visit in 33,138 babies (92.3%), and of these, 2871 (8.66%) were excluded; 495 (17.2%) due to death before CHW’s visit; 581 (20.2%) due to missing assessment data; and 1795 (62.5%) due to first postnatal visit after day 9. Overall, 3592 babies developed first episode neonatal infections during the follow-up period (Fig. 1).

**Fig. 2:**
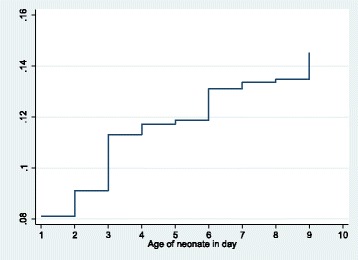
Cumulative incidence of neonatal infections in the first 9 days adjusted for competing risk of deaths

Cumulative incidence of neonatal infections (Fig. 2) in the first 9 days of life was 14.5% (95% CI 14.1–14.9%), and in the first week of life, it was 13.4% (95% CI 12.9–13.8%).

Table 1 summarizes socio-demographic and household factors and their crude association with neonatal infections. Neonates in the lower four household wealth quintiles had increased risk of infections compared to those in the highest wealth quintile. Higher education levels of mothers (≥ primary) significantly decreased the risk of infection in newborns, but father’s education level did not have any significant association. Babies born in families with ≤ 0.5 bedrooms per person (a proxy of crowding) had a significantly higher risk of infection compared to babies in families with > 0.5 bedrooms per person [RR 1.15 (95% CI 1.06–1.25)].

**Table 1:** Association of socio-demographic and household factors with neonatal infections

Characteristics	Neonatal infections in first 2 weeks (*n* = 30,267)				
	Number	Infections	Percent	RR	95% CI
Household wealth quintile					
Lowest quintile (poorest)	6089	755	12.4	1.16	1.04–1.29
Second lowest quintile	6020	749	12.4	1.16	1.05–1.29
Middle quintile	6052	731	12.1	1.12	1.02–1.24
Second highest quintile	6053	744	12.3	1.14	1.03–1.26
Highest quintile (richest)	6053	613	10.1	1.00	
Religion					
Islam	28,934	3445	11.9	1.00	
Others	1333	147	1105	0.98	0.84–1.15
Mother’s age					
< 25 years	9994	1291	12.9	1.00	
25–29 years	10,220	1098	10.71.0	0.85	0.79–0.92
30–34 years	6180	746	12.1	0.97	0.90–1.06
35 years and above	3873	457	11.8	0.96	0.87–1.06
Mother’s education					
Below primary level	15,266	1911	12.5	1.00	
Primary and above	15,001	1681	11.2	0.93	0.87–0.99
Father’s education					
Below primary level	17,607	2147	12.2	1.00	
Primary and above	12,660	1445	11.4	0.98	0.92–1.04
History of child death					
Yes	7499	938	12.5	1.07	0.99–1.15
No	22,768	2654	1179	1.00	
Sleeping room per person					
0.5 or less	24,854	3014	12.1	1.15	1.06–1.25
Higher than 0.5	5413	578	10.7	1.00	

Table 2 shows maternal factors and their association with neonatal infections. Compared to babies born in first pregnancies, third and higher pregnancy order babies had a significantly lower risk of infection. Maternal iron intake for ≥ 60 days during pregnancy was associated with lower risk of neonatal infections (RR 0.91; 95% CI 0.85–0.97) compared to iron intake for < 60 days during pregnancy. Delivery by a skilled attendant significantly lowered the risk of neonatal infections compared to deliveries by unskilled attendants (RR 0.80; 95% CI 0.72–0.90). Home-delivered babies had a higher risk of infection compared to facility-born babies [RR 1.60 (1.37–1.85)]. Those who reported that the birth attendants washed their hands before delivery had a lower risk of infection. Babies whose mothers had retained placenta experienced a significantly higher risk of infection (RR 1.50; 95% CI 1.28–1.75).

**Table 2:** Association of pregnancy and delivery characteristics with neonatal infections

Characteristics	Neonatal infections in first 2 weeks (*n* = 30,267)				
	Number	Infections	Percent	Risk ratio	95% CI
Pregnancy order					
First	5523	729	13.2	1.00	
Second	5831	705	12.1	0.91	0.83–1.00
Third	5187	592	11.4	0.85	0.77–0.94
Fourth or higher	13,726	1566	11.4	0.85	0.79–0.92
Iron consumption during pregnancy					
Less than 60 days	12,349	1597	12.9	1.00	
60 days or more	17,918	1995	11.1	0.91	0.85–0.97
ANC from qualified provider					
Yes	16,056	1867	11.6	1.07	0.99–1.14
No	14,211	1725	12.1	1.00	
Antenatal complications					
Yes	6161	793	12.9	1.07	0.99–1.16
No	24,106	2799	11.6	1.00	
Birth attendant*					
Skilled	3118	269	8.63	0.80	0.72–0.90
Unskilled	27,149	3323	12.2	1.00	
Place of delivery					
Home	27,882	3430	12.3	1.60	1.37–1.85
Facility	2385	162	6.8	1.00	
Washed hands before delivery					
Yes	29,138	3415	11.7	0.83	0.71–0.96
No	1129	177	15.7	1.00	
Prolonged labor					
Yes	2708	328	11.8	1.08	0.97–1.20
No	27,559	3264	12.1	1.00	
Prolonged rupture of membrane					
Yes	2082	276	13.3	1.08	0.96–1.21
No	28,185	3316	11.8	1.00	
Retained placenta					
Yes	753	150	19.9	1.50	1.28–1.75
No	29,514	3442	11.7	1.00	

*Health care providers including physicians, nurses, and paramedics and midwives who have midwifery training for at least 6 months are considered as skilled birth attendants

Table 3 shows newborn factors and their association with neonatal infections. Multiple birth outcome babies had a significantly increased risk of infection [RR 2.47 (95% CI 2.18–2.80)] compared to singleton babies. Lower birth weights significantly increased the risk of infections. Compared to ≥ 2500 g babies, RRs (95% CI) were 6.50 (5.74–7.47), 2.55 (2.29–2.83), and 1.20 (1.12–1.29) for < 1500, 1500–1999, and 2000–2499 g babies, respectively. Asphyxiated babies had 1.87 (95% CI 1.73–2.03) times higher risk of infections than babies with no signs of asphyxia at birth.

**Table 3:** Association of newborn characteristics with neonatal infections

Characteristics	Neonatal infections in first 2 weeks (*n* = 30,267)				
	Number	Infections	Percent	RR	95% CI
Single/multiple birth					
Singleton	29,558	3386	11.5	1.00	
Multiple birth	709	206	29.1	2.47	2.18–2.80
Gestational age at birth					
< 35 weeks	2693	442	16.4	1.44	1.31–1.58
35–36 weeks	3546	415	11.7	1.04	0.95–1.15
37 weeks or more	24,028	2735	11.4	1.00	
Sex of the baby					
Male	15,646	1880	12.0	1.00	
Female	14,621	1712	11.7	0.97	0.92–1.03
Birth weight					
< 1500 g	210	142	67.6	6.50	5.74–7.37
1500–1999 g	1369	357	26.1	2.55	2.29–2.83
2000–2499 g	8386	1040	12.4	1.20	1.12–1.29
2500 g or higher	20,302	2053	10.1	1.00	
Birth asphyxia					
Yes	3427	709	20.7	1.87	1.73–2.03
No	26,840	2883	10.7	1.00	
Sterile cord cutting and tying					
Yes	26,291	3122	11.9	1.08	0.98–1.18
No	3976	470	11.8	1.00	
Non-study substances on cord					
Yes	1779	236	13.3	1.05	0.92–1.20
No	28,488	3356	11.8	1.00	
Dried within 30 min					
Yes	25,719	2893	11.3	1.00	
No	4548	699	15.4	1.18	1.08–1.28
Wrapped within 30 min					
Yes	25,321	2828	11.2	1.00	
No	4946	764	15.5	1.20	1.10–1.31
Breastfed within 1 h					
Yes	18,329	2093	11.4	1.00	
No	11,938	1499	12.6	1.05	0.98–1.13
Oil massaged within 1 h					
Yes	3292	437	13.3	1.11	1.00–1.23
No	26,975	3155	11.7	1.00	
Delayed bath for 1 day					
Yes	26,519	3084	11.6	1.10	0.99–1.21
No	3748	508	13.6	1.00	

Table 4 shows the results from a multivariable GEE log-binomial model. Model 1 is the full model with all covariates, and model 2 is the final model excluding variables based on collinearity and reverse causality. Higher pregnancy order significantly decreased the risk of infections. Compared to the first pregnancy babies, RR (95% CI) of the second, third, and ≥ fourth pregnancy babies were 0.93 (0.85–1.02), 0.88 (0.79–0.97), and 0.79 (0.71–0.87), respectively. Babies born in families with a previous history of child deaths were more likely to develop infections [RR 1.10 (95% CI 1.02–1.19)]. Neonates born in crowded families (≤ 0.5 bedrooms per person) had increased risk of acquiring infections [RR 1.14 (95% CI 1.04–1.25)]. Receipt of antenatal care from qualified providers during pregnancy increased the risk of infections in the neonates [RR 1.11 (95% CI 1.03–1.19)]. Home-born babies had a significantly higher risk of infections compared to facility-born babies [RR 1.86 (95% CI 1.58–2.19)]. Non-sterile cutting and tying of the umbilical cord significantly increased the risk of neonatal infections in the study population [RR 1.15 (95% CI 1.03–1.28)]. Multiple birth babies had a significantly higher risk of infections [RR 1.34 (95% CI: 1.15–1.56)] compared to singleton babies. Lower birth weight significantly increased the risk of infections. Compared to ≥ 2500 g babies, RR (95% CI) for < 1500 g babies was 4.69 (4.01–5.48), 2.15 (1.92–2.42) for 1500–1999 g babies, and 1.15 (1.07–1.25) for 2000–2499 g babies. Babies born with signs of birth asphyxia also had a higher risk of infections [RR 1.65 (95% CI 1.51–1.81)].

**Table 4:** Risk factors of neonatal infections from multivariable GEE log-binomial regression

Risk factors	Model 1 (full)		Model 2 (final)	
	Risk ratio	95% CI	Risk ratio	95% CI
Household wealth quintile (ref: highest quintile)				
Lowest quintile (poorest)	1.05	0.95–1.17	1.04	0.94–1.16
Second lowest quintile	1.06	0.96–1.18	1.06	0.96–1.17
Middle quintile	1.03	0.93–1.14	1.02	0.93–1.14
Second highest quintile	1.08	0.98–1.19	1.07	0.97–1.19
Non-Muslims (ref: Muslim)	0.96	0.84–1.10	0.96	0.84–1.10
Mother’s age (ref: < 25 years)				
25–29 years	0.94	0.87–1.02	0.94	0.87–1.03
30–34 years	1.10	1.00–1.21	1.10	1.00–1.21
35 years and above	1.08	0.96–1.22	1.09	0.96–1.23
Mothers primary or above (ref: below primary)	0.94	0.87–1.02	0.94	0.87–1.02
Father’s primary and above (ref: below primary)	1.08	1.00–1.16	1.08	1.00–1.16
Pregnancy order (ref: first pregnancies)				
Second	0.93	0.85–1.02	0.93	0.85–1.02
Third	0.88	0.79–0.97	0.88	0.79–0.97
Fourth or higher	0.79	0.71–0.87	0.79	0.71–0.87
History of child death (ref: no history of death)	1.10	1.02–1.19	1.10	1.02–1.19
≤ 0.5 room per person in the house (ref: > 0.5)	1.14	1.04–1.26	1.14	1.04–1.25
Antenatal iron consumption for ≥ 60 days (ref: < 60 days)	0.96	0.89–1.03	0.96	0.89–1.03
ANC from qualified provider (ref: no)	1.11	1.03–1.19	1.11	1.03–1.19
Antenatal complications (ref: no complications)	1.01	0.92–1.10	1.01	0.92–1.10
Home delivery (ref: facility delivery)	2.13	1.73–2.62	1.86	1.58–2.19
Skilled birth attendant (ref: unskilled attendant)	1.19	1.01–1.40		
Washed hands before delivery (ref: no)	0.92	0.77–1.09		
Prolonged labor (ref: no)	1.04	0.93–1.16	1.04	0.94–1.16
Prolonged rupture of membrane (ref: no)	1.02	0.91–1.14	1.02	0.92–1.14
Retained placenta (ref: no)	1.15	0.98–1.35	1.14	0.96–1.34
Non-study substances on cord (ref: no)	0.96	0.85–1.09	0.96	0.85–1.09
Non-sterile cord cutting and tying (ref: sterile)	1.13	1.02–1.25	1.15	1.03–1.28
Multiple birth (ref: singleton birth)	1.33	1.15–1.55	1.34	1.15–1.56
Female baby (ref: male baby)	0.96	0.90–1.02	0.96	0.89–1.02
Gestational age at birth (ref: ≥ 37 weeks)				
< 35 weeks	1.09	0.99–1.19	1.08	0.99–1.19
35–36 weeks	0.97	0.87–1.07	0.96	087–1.07
Birth weight (ref: ≥ 2500 g)				
< 1500 g	4.70	4.01–5.51	4.69	4.01–5.48
1500–1999 g	2.15	1.92–2.41	2.15	1.92–2.42
2000–2499 g	1.16	1.07–1.25	1.15	1.07–1.25
Birth asphyxia (ref: no birth asphyxia)	1.66	1.51–1.82	1.65	1.51–1.81
Dried baby after 30 min (ref: < 30 min)	0.93	0.66–1.30	0.94	0.73–1.20
Wrapped after 30 min (ref: < 30 min)	1.20	0.96–1.51	1.19	0.95–1.49
Breastfed after 1 h (ref: within 1 h)	0.96	0.84–1.09		
Oil massaged within 1 h (ref: no massage within 1 h)	1.03	0.91–1.16		
Bathed baby within 1 day (ref: delayed bath for 1 day)	1.01	0.78–1.32		

## Discussions

We report high cumulative incidence (14.5%) of infections as ascertained by CHWs in four scheduled postnatal visits in the first 9 days of life in our study population. We also demonstrate that multiple socio-demographic, household, maternal, and newborn characteristics are significantly associated with neonatal infections. These data come from a well-defined large (*n* = 30,267) population-based birth cohort in a rural area of Bangladesh. Comparison between estimates of clinical neonatal infections estimated in different studies is challenging due to the use of variable clinical algorithms, duration of follow-up, and frequency of assessment. Our estimate is higher than the previous estimates from the same setting. Baqui et al. (2009) reported 5.6% incidence of very severe disease and 11.2% possible very severe disease in the first week of life from this study area [28]. Although the estimates appear similar, the duration of follow-up, frequency of assessment, and clinical algorithms are different. Estimates from India, Nepal, and Pakistan used a similar clinical algorithm but used variable follow-up duration and assessment schedule ranges from 5.0 to 11.0% [29].

Our data shows that higher birth order decreased the risk of neonatal infections, which is consistent with the findings from other studies. One study from Sweden reported an odds ratio of 0.56 (95% CI 0.45–0.70) for multiparity compared to primiparity [30]. Another study in Nepal found babies born to primipara mothers had a higher risk of infection (OR 1.58) compared to babies of multipara mothers [31]. These findings are likely to occur due to the improved newborn care practices by experienced mothers, particularly early initiation of breastfeeding [32].

Previous death of a child in the family is an established risk factor for child death [33]. However, reports on its association with immediate contributors of neonatal deaths are mixed. One study in Nepal did not find any association between previous child deaths and asphyxia-related neonatal death [34]; however, other studies showed its association with preterm birth or small for gestational age [35, 36]. With this study, now we show that previous child death is associated with early neonatal infections. Density of people in the household has also been shown to increase the risk of child mortality in different settings [37, 38]. Our data show that < 1 room per two persons significantly increased the risk of neonatal infections. Increased density of people in the household represents overcrowding, which contributes to the transmission of infections through respiratory droplets [39, 40]. We found an increased risk of infections in babies born to mothers who received at least one antenatal care (ANC) visit from a qualified provider compared to babies whose mothers did not receive any ANC visits from qualified providers. This finding contradicts the generally held belief that ANC reduces the risk of neonatal infections and mortality [41]. This could have resulted from misclassification between routine ANC and care seeking for antenatal complications, as both data are collected as maternal reports. Home-delivered newborns were at greater risk of developing infections compared to facility-born babies in our study population, which is consistent with the findings from other studies [42, 43]. Although home-born babies received their first assessment visits by CHWs much earlier than the hospital-born babies (median age at first visit in home-delivered babies 15 h compared to 78 h in facility-born babies), the association remained significant even after adjusting for age at first visit. Prolonged duration of labor and prolonged rupture of membrane (PROM) were not associated with neonatal infections, although others have found that these conditions are associated with newborn infection [44, 45]. Failure to cut and tie the umbilical cord aseptically increased the risk of infections significantly. This supports that the clean cord practice can prevent neonatal infections and deaths in settings where most births occur at home [46]. In this study, babies born in multiple births were at higher risk of infections compared to singleton babies, which is consistent with the reports in previous studies [47]. We did not find any elevated risk associated with male sex as reported in earlier studies [31, 48]. Gestational age at birth was not associated with neonatal infections in the adjusted model, but birth weight was highly associated, although both preterm and low birth weight are established risk factors for neonatal infections [49]. Birth asphyxia is significantly associated with infections, as was also reported in the previous studies [49, 50].

This study has several strengths. This is a large population-based study in a developing country setting with routine pregnancy and birth surveillance in place. The study enrolled a large number of newborns allowing adequate sample size for estimating the incidence of neonatal infections with high precision. The study also provides adequate samples for testing hypotheses for smaller associations with high power. All risk factors were measured before the occurrence of outcomes that allowed eliminating certain biases that are common in cross-sectional studies. We adjusted for survival bias allowing late entry at the age of health workers’ first assessment and competing risk of deaths for estimating cumulative incidence function of infections.

The study also had several limitations. The algorithm used for the diagnosis of neonatal infections may have high sensitivity and low specificity, and the clinical algorithm used to ascertain newborn infections overlaps with prematurity, LBW, and birth asphyxia [51]. Laboratory diagnosis of infection was not performed in this study. Although the presence of interventions may have influenced the incidence of infections, the analysis of risk factors was adjusted for intervention effects. This study captured infections in the first 2 weeks of life; thus, the findings do not represent infections in the entire neonatal period. However, this is the most vulnerable period accounting for almost 90% of neonatal deaths in developing countries [52]. The infants who died before the CHW’s first assessment were excluded from this study, resulting in left truncation and residual survival bias.

Several areas for future research are highlighted with the findings of this study. Similar studies should be conducted to capture the entire neonatal period to generate further population-based data on burden and risk factors of neonatal infections in low- and middle-income countries. The effect of essential neonatal care on late-onset infections also needs to be examined. Another area of future interest is the incidence of recurrent infections in the neonatal period and within early infancy period (i.e., the first 2 months of life).

## Conclusions

In conclusion, the high burden of neonatal infections remains a major challenge to reducing neonatal deaths in Bangladesh. Several socio-economic, household, maternal, and newborn factors were shown to increase the risk of neonatal infection. About half of the cases developed signs very early on the day of birth. Two thirds of home births in Bangladesh with almost negligible outreach worker visitation coverage [53] remain as a huge challenge in terms of identification of the danger signs, early on. Thus maternal and child health programs in low- and middle-income countries should design strategies to promote preventive measures, identify and manage newborns with clinical infections at the community and first level facility, and promote facility delivery in the long run. The Ministry of Health and Family Welfare (MOH&FW) in Bangladesh is about to launch its 4th Health, Nutrition and Population (HNP) Sector program, with a strong National Newborn Health Program (NNHP) inbuilt in it [54]. In the context of wider geographic disparity in newborn mortality in the country [53], Bangladesh needs differential program design. Keeping in alignment with recommendations from the Bangladesh Every Newborn Action Plan (BENAP) [55], the NNHP puts a strong emphasis on a comprehensive social and behavioral change communication strategy focused towards changing community norms and behavior relevant to newborn care. To be optimally effective, both the differential approach and a customized approach for SBCC strategy should draw on the risk factors identified in this study.

## Abbreviations

BNCP: Birth and newborn care preparedness
CHW: Community health worker
CI: Confidence interval
GEE: Generalized estimating equation
IMCI: Integrated Management of Childhood Illness
LBW: Low birth weight
MWRA: Married woman of reproductive age
OR: Odds ratio
RR: Risk ratio
WHO: World Health Organization
